# CircTM7SF3 contributes to oxidized low-density lipoprotein-induced apoptosis, inflammation and oxidative stress through targeting miR-206/ASPH axis in atherosclerosis cell model in vitro

**DOI:** 10.1186/s12872-020-01800-x

**Published:** 2021-02-02

**Authors:** Xiaojuan Wang, Ming Bai

**Affiliations:** Department of Heart Center, The First Hospital of Lanzhou University, No.1 Donggang West Road, Chengguan District, Lanzhou, 730030 Gansu China

**Keywords:** Atherosclerosis, Ox-LDL, circTM7SF3, miR-206, ASPH

## Abstract

**Background:**

Atherosclerosis (AS) is a chronic inflammatory disorder. The aim of our study was to explore the role of circular RNA (circRNA) transmembrane 7 superfamily member 3 (circTM7SF3) in AS progression.

**Methods:**

Experiments were conducted using oxidized low-density lipoprotein (ox-LDL)-induced THP-1-derived macrophages and differentiated human monocyte-derived macrophages (hMDMs). Quantitative real-time polymerase chain reaction (qRT-PCR) was used to detect the expression of circTM7SF3, its linear form TM7SF3, microRNA-206 (miR-206) and aspartyl (asparaginyl) β-hydroxylase (ASPH) messenger RNA (mRNA). Cell viability and apoptosis were examined by 3-(4,5-Dimethylthiazol-2-yl)-2,5-diphenyltetrazolium bromide (MTT) assay and flow cytometry. Cell inflammation was analyzed by measuring the production of tumor necrosis factor α (TNF-α) and interleukin 6 (IL-6) using enzyme-linked immunosorbent assay (ELISA) kits. Cell oxidative stress was assessed through analyzing the levels of oxidative stress markers using their corresponding commercial kits. Dual-luciferase reporter assay and RNA-pull down assay were used to confirm the interaction between miR-206 and circTM7SF3 or ASPH. The protein level of ASPH was examined by Western blot assay.

**Results:**

CircTM7SF3 level was markedly increased in the serum samples of AS patients and ox-LDL-induced THP-1-derived macrophages compared with their matching counterparts. ox-LDL induced-damage in THP-1 cells was partly attenuated by the interference of circTM7SF3. MiR-206 was a downstream molecular target of circTM7SF3. Si-circTM7SF3-mediated effects in ox-LDL-induced THP-1-derived macrophages were partly ameliorated by the addition of anti-miR-206. MiR-206 directly interacted with ASPH mRNA. CircTM7SF3 silencing reduced the expression of ASPH partly through up-regulating miR-206 in THP-1-derived macrophages. ASPH overexpression partly counteracted the effects induced by miR-206 overexpression in ox-LDL-induced THP-1-derived macrophages.

**Conclusion:**

CircTM7SF3 contributed to ox-LDL-induced injury in AS cell model through up-regulating the expression of ASPH via targeting miR-206.

## Background

Atherosclerosis (AS) is a chronic inflammatory disorder, and it causes cardiovascular diseases, including stroke and myocardial infarction [1, 2]. Macrophages play crucial function in AS initiation and progression, and the abnormal apoptosis and failure in the scavenging of apoptotic body both contribute to the inflammatory response of AS [3]. The pathogenesis of AS needs to be explored to find novel effective targets for AS treatment. Oxidized low-density lipoprotein (ox-LDL) induces the inflammatory response of macrophages, eventually causing the apoptosis of macrophages [4]. We established the AS cell model through treating THP-1-derived macrophages by ox-LDL to study the molecular mechanism behind AS progression in vitro [5].

Circular RNAs (circRNAs) exert important roles in many cardiovascular diseases and cancers [6, 7]. CircCHFR contributed to the proliferation and migration of vascular smooth muscle cells (VSMCs) through targeting miR-370/FOXO1/Cyclin D1 axis in AS [8]. Circ_0003575 suppressed the proliferation and angiogenesis in ox-LDL-induced HUVECs [9]. CircRNA transmembrane 7 superfamily member 3 (circTM7SF3) level was aberrantly enhanced in differentiated THP-1 cells treated with ox-LDL according to GSE107522 dataset [10]. However, the function of circTM7SF3 in AS remains to be illustrated.

Accumulating studies have pointed out the pivotal roles of microRNAs (miRNAs) in human diseases, including AS [11–13]. The regulatory roles of miR-206 in thyroid cancer, lung cancer, breast cancer, prostate cancer and colon cancer have been uncovered by former articles [14–18]. Long non-coding RNA (lncRNA) UCA1 was reported to accelerate ox-LDL-induced oxidative stress and apoptosis of THP-1 cells through sponging miR-206 [19]. Nevertheless, the interaction between miR-206 and circTM7SF3 has not been reported.

Aspartyl (asparaginyl) β-hydroxylase (ASPH) belongs to type II transmembrane protein family [20]. Recent studies have pointed out the crucial regulatory role of ASPH in the initiation and development of cancers. For instance, miR-135a was found to suppress the progression of endometrial cancer through targeting ASPH [21]. MiR-200a restrained the proliferation and motility of hepatocellular carcinoma cells through reducing ASPH level [22]. However, the interaction between miR-206 and ASPH has never been identified and the role of ASPH in AS remains to be disclosed.

Our purpose was to explore the biological function and working mechanism of circTM7SF3 in AS development through using ox-LDL-induced AS cell model. The interaction between miR-206 and circTM7SF3 or ASPH was identified for the first time in our study.

## Methods

### Sample collection

Thirty-nine peripheral blood samples (2 mL per subject) obtained from AS patients, along with 39 peripheral blood samples (2 mL per subject) obtained from healthy volunteers were collected at The First Hospital of Lanzhou University. Written informed consent was provided by each subject before sample collection. The protocol in this study was approved by the Ethics Committee of The First Hospital of Lanzhou University. Clinical and biological characteristics of human participants were shown in Table 1.

**Table 1:** Clinical and biological characteristics of human participants

Characteristics	AS patients (*n* = 39)	Healthy participants (*n* = 39)	*P* value	Statistical method
Age (year)	52.26 ± 6.39	54.68 ± 7.45	0.1278	Student’s *t*-test
Male, n (%)	27 (69.23)	28 (71.79)	0.8039	χ^2^ test
BMI (kg/m^2^)	24.52 ± 1.46	23.96 ± 1.42	0.0900	Student’s *t*-test
Smoking, n (%)	24 (61.53)	26 (66.67)	0.6369	χ^2^ test
Hypertension, n (%)	30 (76.92)	12 (30.77)	< 0.001	χ^2^ test
Diabetes, n (%)	7 (17.95)	4 (10.25)	0.3291	χ^2^ test
Hyperlipidemia, n (%)	28 (71.79)	26 (66.67)	0.6237	χ^2^ test
TG (mmol/L)	2.06 ± 0.68	1.82 ± 0.56	0.0930	Student’s *t*-test
TC (mmol/L)	5.08 ± 0.62	4.83 ± 0.52	0.0574	Student’s *t*-test
LDL-L (mmol/L)	3.13 ± 0.59	2.89 ± 0.49	0.0544	Student’s *t*-test
HDL-L (mmol/L)	1.04 ± 0.21	1.09 ± 0.18	0.2625	Student’s *t*-test
WBC (10^9^/L)	7.15 ± 2.01	6.84 ± 1.94	0.4904	Student’s *t*-test

*BMI* body mass index, *TG* triglycerides, *TC* totalcholesterol, *LDL-C* low-density lipoprotein cholesterol, *HDL-C* high-density lipoprotein cholesterol, *WBC* white blood cells

### AS cell model in vitro

Human acute monocytic leukemia cell line THP-1 was purchased from BeNa Culture Collection (Beijing, China; Cat. no. BNCC288554). THP-1 cells were maintained in Roswell Park Memorial Institute-1640 medium (RPMI-1640, Gibco, Carlsbad, CA, USA; Cat. no. 12633012) supplemented with 10% fetal bovine serum (FBS; Gibco; Cat. no. 16140071) and 10% penicillin/streptomycin antibiotic reagent. Cells were cultivated in a humidified incubator at 37 °C with 5% CO_2_.

10 nM phorbol 12-myristate 13-acetate (PMA; Sigma, St. Louis, MO, USA; Cat. no. 19-144) was used to differentiate THP-1 to macrophages [23]. AS cell model was established through exposing differentiated THP-1 cells to 50 μg/mL ox-LDL for 24 h.

Human monocyte-derived macrophages (hMDMs) were extracted from the peripheral blood of one volunteer using the methods described previously [24, 25]. hMDMs were cultivated using RPMI-1640 (Gibco) added with 10% FBS (Gibco) and 200 nM PMA (Sigma) for 72 h. Differentiated hMDMs were washed using phosphate buffer saline (PBS; Sangon Biotech, Shanghai, China) for three times and then induced by 50 μg/mL ox-LDL for 24 h for further analysis.

### RNA extraction and quantitative real-time polymerase chain reaction (qRT-PCR)

RNA samples from blood samples, THP-1-derived macrophages and differentiated hMDMs were isolated using TRIzol reagent (Invitrogen, Carlsbad, CA, USA; Cat. no. 15596018). The complementary DNA (cDNA) of miR-206 was synthesized using TaqMan MicroRNA Reverse Transcription kit (Applied Biosystems, Rotkreuz, Switzerland; Cat. no. 4366597). The reverse transcription of circTM7SF3, TM7SF3 and ASPH was conducted with High-Capacity cDNA Reverse Transcription kit (Applied Biosystems; Cat. no. 4374966). TB Green® Premix Ex Taq™ kit (Takara, Dalian, China; Cat. no. RR420L) and specific primers were used for PCR reaction. U6 for miR-206 and glyceraldehyde-3-phosphate dehydrogenase (GAPDH) for circTM7SF3, TM7SF3 and ASPH were used as house-keeping genes. Specific primer sequences were listed in Table 2. Fold change of circTM7SF3, TM7SF3, miR-206 and ASPH was calculated by the 2^−ΔΔCt^ method.

**Table 2:** Primers in qRT-PCR

Gene	Species	Forward primer	Reverse primer
circTM7SF3 TM7SF3 miR-206 ASPH U6 GAPDH	human human human human human human	GCAGGAGAGGGTAGTTGTGC AGCGAGGGTCTTATTGAATT GATTCGCCAAAGGAAATAGC GTTACCACGTGGAAGAGAC AGGGGCCGGACTCGTCATACT CGGGAAACTGTGGCGTGATG	TCCCCAAGTACCAAGTGCAT GGAATTGGAAAGGAGAGTCG GTTACAAGGTCATCCAAGAC GCTTGTTCCTCATAGACTTG GGCGGCACCACCATGTACCCT ATGACCTTGCCCACAGCCTT

### Stability analysis of circTM7SF3

Transcription inhibitor Actinomycin D (Sigma; 2 mg/mL; Cat. no. SBR00013) was incubated with THP-1-derived macrophages for 0 h, 4 h, 8 h, 12 h or 24 h, and qRT-PCR was conducted to detect the expression of circTM7SF3 and TM7SF3 messenger RNA (mRNA).

A total of 3 μg RNA sample isolated from THP-1-derived macrophages was incubated with or without 9 units RNase R (Sigma; Cat. no. R6513) for 30 min at room temperature, and the levels of circTM7SF3 and TM7SF3 mRNA were examined by qRT-PCR.

### Cell transfection

CircTM7SF3 specific small interfering RNAs (100 nM), including si-circTM7SF3_1 (5′-UGGAUUUGUACCAUUCUUCUG-3′), si-circTM7SF3_2 (5′-ACUCAUUGGUUCCUUUAAGGG-3′) and si-circTM7SF3_3 (5′-UCAUAUUCUGAAUCUCAUCCU-3′), siRNA negative control (si-NC; 100 nM), miR-206 (40 nM), miR-NC (40 nM), miR-206 inhibitor (anti-miR-206; 20 nM), anti-miR-NC (20 nM), ASPH ectopic expression plasmid (pcDNA-ASPH; 1 μg) and pcDNA-NC (1 μg) from Sangon Biotech were transfected into differentiated THP-1 cells or differentiated hMDMs in 6-well plates using Lipofectamine® 2000 reagent (Invitrogen; Cat. no. 11668019).

### 3-(4,5-Dimethylthiazol-2-yl)-2,5-diphenyltetrazolium bromide (MTT) assay

Cell viability was analyzed by MTT colorimetric assay. Briefly, 20 μL 2.5 mg/mL MTT solution (Invitrogen; Cat. no. M6494) was added into the wells of 96-well plates, and THP-1-derived macrophages were incubated with MTT solution for 4 h at 37 °C. After that, cell supernatant was discarded and the formazan product was dissolved through adding 150 μL dimethyl sulfoxide (DMSO). The optical density (OD) value (570 nm) was measured using a microplate reader (Bio-Rad, Shanghai, China).

### Flow cytometry

Annexin V-fluorescein isothiocyanate (FITC)/propidium iodide (PI) apoptosis detection kit (R&D Systems, Minneapolis, MN, USA; Cat. no. 4817-60-K) was used to analyze the apoptotic rate of differentiated THP-1 cells. Differentiated THP-1 cells were collected and mixed with 100 μL Annexin V-FITC/PI reagent for 20 min in a dark room to mark the phosphatidylserine and DNA content. The apoptotic THP-1 cells at early stage (FITC^+^/PI^−^; the first quadrant) and late stage (FITC^+^/PI^+^; the fourth quadrant) were identified and the apoptotic rate was analyzed by flow cytometry.

### Enzyme-linked immunosorbent assay (ELISA)

Tumor necrosis factor α (TNF-α) Quantikine ELISA Kit (R&D Systems; Cat. no. DTA00D) and interleukin 6 (IL-6) Quantikine ELISA Kit (R&D Systems; Cat. no. D6050) were used in this experiment. The supernatant of THP-1-derived macrophages and differentiated hMDMs was collected to analyze the concentrations of TNF-α and IL-6.

### Oxidative stress status analysis

The oxidative stress status of THP-1 cells was analyzed through measuring several oxidative indicators. The levels of reactive oxygen species (ROS), superoxide dismutase (SOD), malondialdehyde (MDA) and inducible nitric oxide synthase (iNOS) were measured by ROS Assay Kit (Beyotime, Shanghai, China; Cat. no. S0033M), SOD Activity Assay Kit (BioVision, Milpitas, CA, USA; Cat. no. K335), MDA Assay Kit (Beyotime; Cat. no. S0131M) and iNOS kit (Abcam, Cambridge, MA, USA; Cat. no. ab253217), respectively.

### Establishment of circRNA-miRNA-mRNA signal axis

The interaction between circTM7SF3 and miR-206 was predicted by starbase software. The mRNA targets of miR-206 were predicted by starbase, miRWalk and TargetScan softwares.

### Dual-luciferase reporter assay

The predicted miR-206 binding sites in circTM7SF3 were amplified and cloned into psiCHECK-2 plasmid (Promega, Madison, WI, USA; Cat. no. C8021) to generate circTM7SF3-wild type (WT). The wild-type sequence of ASPH was also cloned into psiCHECK-2 plasmid (Promega) to generate ASPH 3′ untranslated region (3’UTR)-WT. The mutant luciferase plasmids (circTM7SF3-mutant type (MUT) and ASPH 3’UTR-MUT) were constructed through inserting mutant sequences (shown in Fig. 3a and 5c) into psiCHECK-2 plasmid (Promega) using Site-directed gene mutagenesis kit (Agilent Technologies, Santa Clara, CA, USA; Cat. no. 200518). THP-1 cells in 24-well plates were co-transfected with 20 nM miR-206 or miR-NC and 100 ng re-constructed plasmids. After 48-h transfection, luciferase activities in different groups were detected using the dual-luciferase assay kit (Promega; Cat. no. E1910).

### RNA-pull down assay

All procedures were conducted under the RNase-free condition. THP-1-derived macrophages were disrupted, and 2 μg cell lysate was incubated with 100 pmol biotinylated RNA (Bio-miR-NC, Bio-miR-206 and Bio-miR-206-MUT) followed by 100 μL streptavidin agarose beads incubation for 1 h. After boiling using sodium dodecyl sulfate (SDS) buffer, qRT-PCR was applied to detect the enrichment of circTM7SF3 and ASPH mRNA.

### Western blot assay

Total protein samples from THP-1-derived macrophages were extracted with cell lysis buffer (Abcam; Cat. no. ab152163) added with protease inhibitors (Roche, Basel, Switzerland; Cat. no. 04693132001). Equal amounts of protein samples (30 μg) were separated by Bio-Rad Bis-Tris Gel system (Bio-Rad) and electro-transferred to polyvinylidene fluoride (PVDF) membranes (Millipore, Billerica, MA, USA; Cat. no. IPVH00010). Primary antibodies, including anti-ASPH (Sigma; Cat. no. SAB1402121) at the dilution of 1:5000 and anti-GAPDH (Sigma; Cat. no. SAB2701826) at the dilution of 1:20000, were incubated with PVDF membranes at 4 °C overnight. After washing, the PVDF membranes were incubated with secondary antibody (Sigma; Cat. no. A3687) at the dilution of 1:5000. After washing, the intensities of protein bands were visualized by enhanced chemiluminescent visualization (ECL) system (Pierce, Rockford, IL, USA; Cat. no. 32106).

### Statistical analysis

The statistical results from three independent experiments with three technical replications were displayed as mean ± standard deviation. The differences in two groups were analyzed by Student’s *t*-test, and the comparison in multiple groups was analyzed by one-way analysis of variance (ANOVA) followed by Tukey’s test. Linear correlation was analyzed by Spearman’s correlation coefficient. Differences were statistically significant with the *P* value of less than 0.05.

## Results

### CircTM7SF3 level is enhanced in THP-1-derived macrophages by ox-LDL treatment

**Fig. 1:**
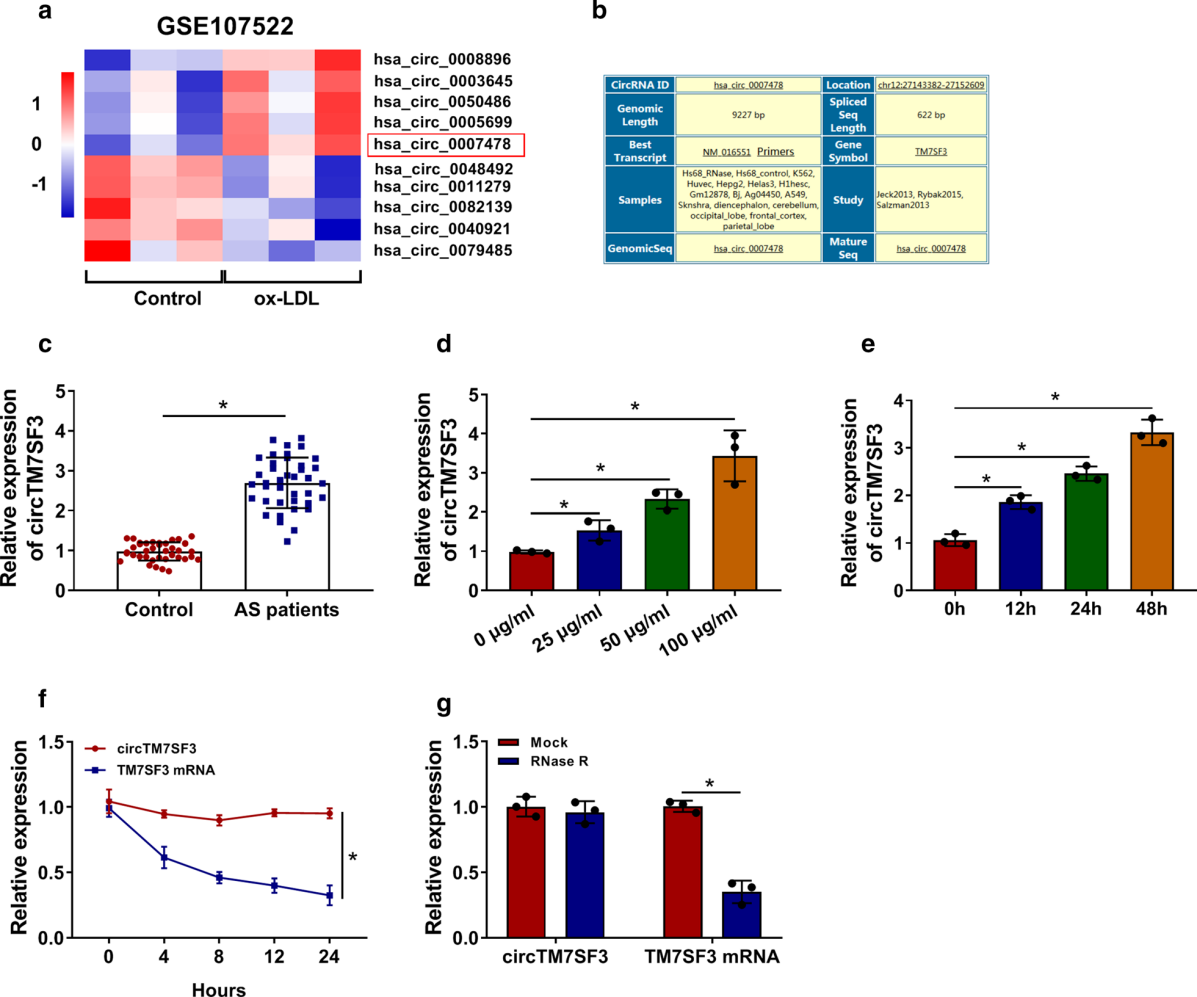
CircTM7SF3 level is enhanced in THP-1-derived macrophages by ox-LDL treatment. **a** The five most up-regulated and down-regulated circRNAs in THP-1-derived macrophages after ox-LDL treatment according to GSE107522 dataset were shown. **b** The gene information of circTM7SF3 from Circular RNA Interactome (https://circinteractome.nia.nih.gov) was shown. **c** CircTM7SF3 expression in the serum of Control patients (*n* = 39) and AS patients (*n* = 39) was detected by qRT-PCR. The results were analyzed using Student’s *t*-test. **d** THP-1 cells (*n* = 3) were exposed to increased doses of ox-LDL for 24 h, and TM7SF3 level was detected by qRT-PCR. The results were assessed by ANOVA followed by Tukey’s test. **e** qRT-PCR was applied to measure the enrichment of TM7SF3 in THP-1 cells (*n* = 3) treated with 50 μg/mL ox-LDL for fixed time interval. The results were assessed by ANOVA followed by Tukey’s test. **f** After treating with Actinomycin D for 0 h, 4 h, 8 h, 12 h or 24 h, RNA was isolated from THP-1 cells (*n* = 3), and the expression of circTM7SF3 and TM7SF3 mRNA was detected by qRT-PCR. The results were analyzed using Student’s *t*-test. **g** RNA samples isolated from THP-1 cells (*n* = 3) were grouped into Mock group and RNase R group, and the levels of circTM7SF3 and TM7SF3 mRNA were analyzed by qRT-PCR. The results were analyzed using Student’s *t*-test. *: *P* < 0.05

According to the results of GSE107522, the five most up-regulated and down-regulated circRNAs in THP-1-derived macrophages with ox-LDL treatment compared with un-treated THP-1-derived macrophages were shown in Fig. 1a. CircTM7SF3 (hsa_circ_0007478) was one of the up-regulated circRNAs after ox-LDL treatment in THP-1-derived macrophages (Fig. 1a). The gene information of circTM7SF3 in Circular RNA Interactome database (https://circinteractome.nia.nih.gov) was shown in Fig. 1b. Serum samples from healthy volunteers (Control group; *n* = 39) and AS patients (*n* = 39) were collected to explore if circTM7SF3 was up-regulated in AS patients. A significant up-regulation in the expression of circTM7SF3 was observed in the serum samples of AS patients compared with that of control group (Fig. 1c). The clinical and biological characteristics of human participants were listed in Table 1, and patients with AS were significantly associated with hypertension (*P* < 0.001). The regulatory relationship between circTM7SF3 and ox-LDL in THP-1-derived macrophages was subsequently tested. As shown in Fig. 1d and e, ox-LDL treatment time- or dose-dependently increased the expression of circTM7SF3 in THP-1-derived macrophages. 50 μg/mL ox-LDL treatment for 24 h was used in the following experiments. The stability of circTM7SF3 was tested using transcription inhibitor Actinomycin D and exonuclease RNase R. Compared with its linear counterpart TM7SF3 mRNA, circTM7SF3 level remained stable after Actinomycin D treatment for 24 h (Fig. 1f). TM7SF3 mRNA was significantly decreased with RNase R treatment, and circTM7SF3 was resistant to RNase R digestion (Fig. 1g).

### Ox-LDL suppresses the viability and promotes the apoptosis, inflammation and oxidative stress of THP-1-derived macrophages through elevating circTM7SF3 level

**Fig. 2:**
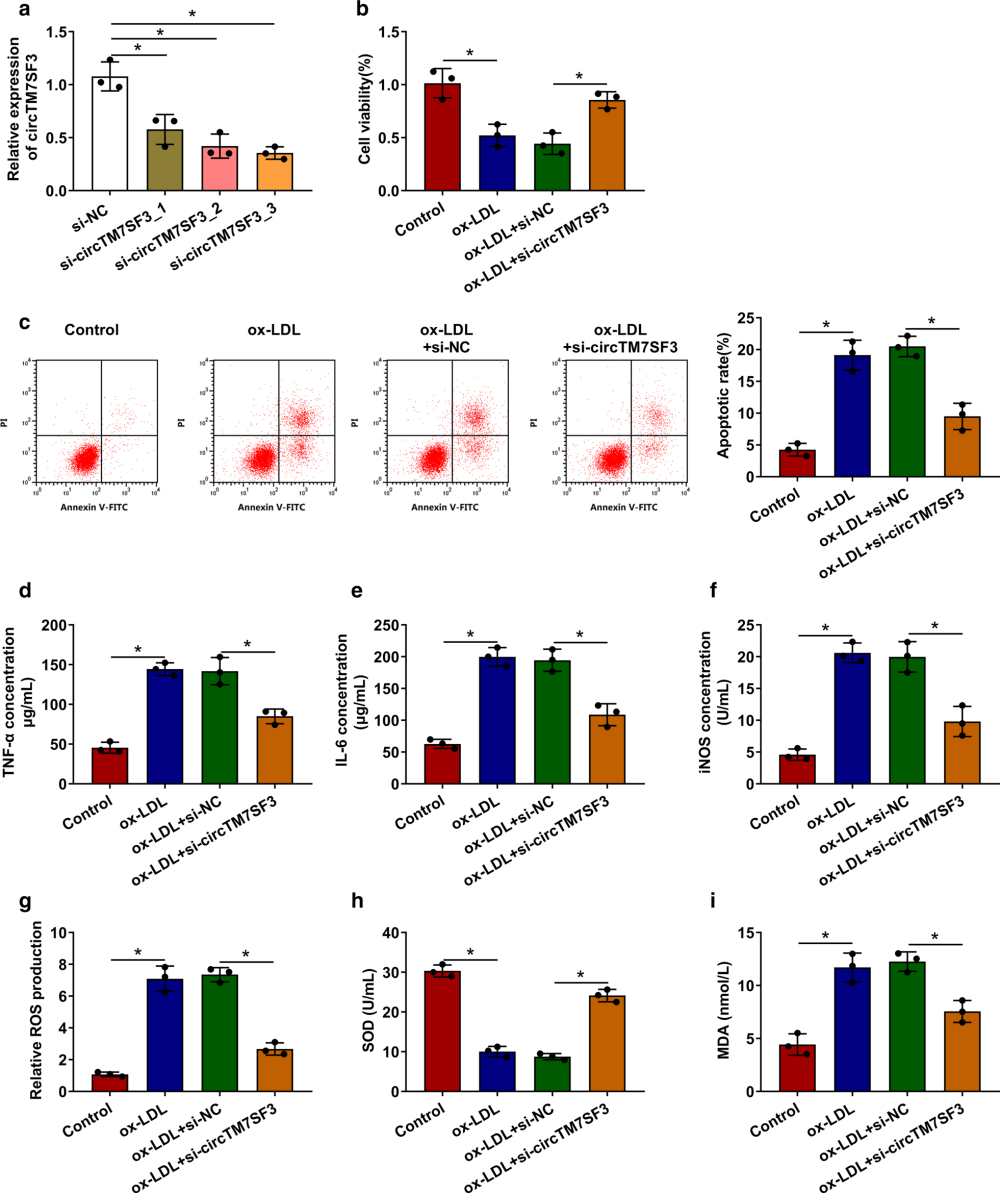
ox-LDL suppresses the viability and promotes the apoptosis, inflammation and oxidative stress of THP-1-derived macrophages through elevating circTM7SF3 level. **a** CircTM7SF3 level was examined in THP-1 cells (*n* = 3) transfected with three siRNAs targeting circTM7SF3 by qRT-PCR. Si-circTM7SF3_3 was written as si-circTM7SF3 in the later assays. The results were assessed by ANOVA followed by Tukey’s test. (B-I) THP-1 cells were incorporated into four groups: Control, ox-LDL, ox-LDL + si-NC and ox-LDL + si-circTM7SF3. The results in B-I were assessed by ANOVA followed by Tukey’s test. **b** Cell viability was assessed using MTT assay (*n* = 3). **c** Cell apoptosis was evaluated by flow cytometry (*n* = 3). **d** and **e** Inflammatory response was detected through examining the production of TNF-α and IL-6 via ELISA kits (*n* = 3). **f**-**i** The oxidative stress was analyzed through measuring iNOS, ROS, SOD and MDA by their corresponding kits (*n* = 3). *: *P* < 0.05

We silenced circTM7SF3 in ox-LDL-induced THP-1-derived macrophages to explore if the up-regulation of circTM7SF3 was necessary for ox-LDL-induced effects in THP-1-derived macrophages. Three siRNAs targeting circTM7SF3 were designed to silence circTM7SF3 in THP-1-derived macrophages. Among three siRNAs, the knockdown efficiency of si-circTM7SF3_3 was the highest (Fig. 2a), thus si-circTM7SF3_3 (written as si-circTM7SF3) was selected in the following loss-of-function experiments. ox-LDL down-regulated cell viability and promoted the apoptosis of THP-1-derived macrophages, while the interference of circTM7SF3 largely recovered the viability and inhibited the apoptosis of THP-1-derived macrophages (Figu. 2 b and c). We also detected the concentrations of inflammation-associated cytokines to assess cell inflammation. ox-LDL-induced elevation of TNF-α and IL-6 was partly attenuated by the silencing of circTM7SF3 in THP-1-derived macrophages (Fig. 2d and e). To further verify the effect of circTM7SF3 interference on ox-LDL-induced inflammation in AS, primary human monocyte-derived macrophages (hMDMs) were isolated from the peripheral blood of one volunteer, and we treated hMDMs with PMA followed by ox-LDL to establish AS cell model. As shown in Additional file 1: Figure 1A, ox-LDL-induced up-regulation in circTM7SF3 expression was partly counteracted by the addition of si-circTM7SF3 in differentiated hMDMs. ox-LDL treatment promoted the secretion of inflammatory cytokines (TNF-α and IL-6), while these promoting effects were partly attenuated by the interference of circTM7SF3 in differentiated hMDMs (Additional file 1: Figure 1B and C), which demonstrated that ox-LDL induced inflammatory response in differentiated hMDMs partly through up-regulating circTM7SF3. We also assessed the oxidative stress status of THP-1 cells through measuring the levels of several oxidative stress indicators (iNOS, ROS, SOD and MDA). ox-LDL-induced up-regulation of iNOS, ROS and MDA and down-regulation of SOD were partly overturned by circTM7SF3 knockdown in THP-1-derived macrophages (Fig. 2f-i), suggesting that ox-LDL-induced oxidative stress in THP-1-derived macrophages was partly attenuated by the interference of circTM7SF3.

### CircTM7SF3 directly interacts with miR-206 in THP-1-derived macrophages

**Fig. 3:**
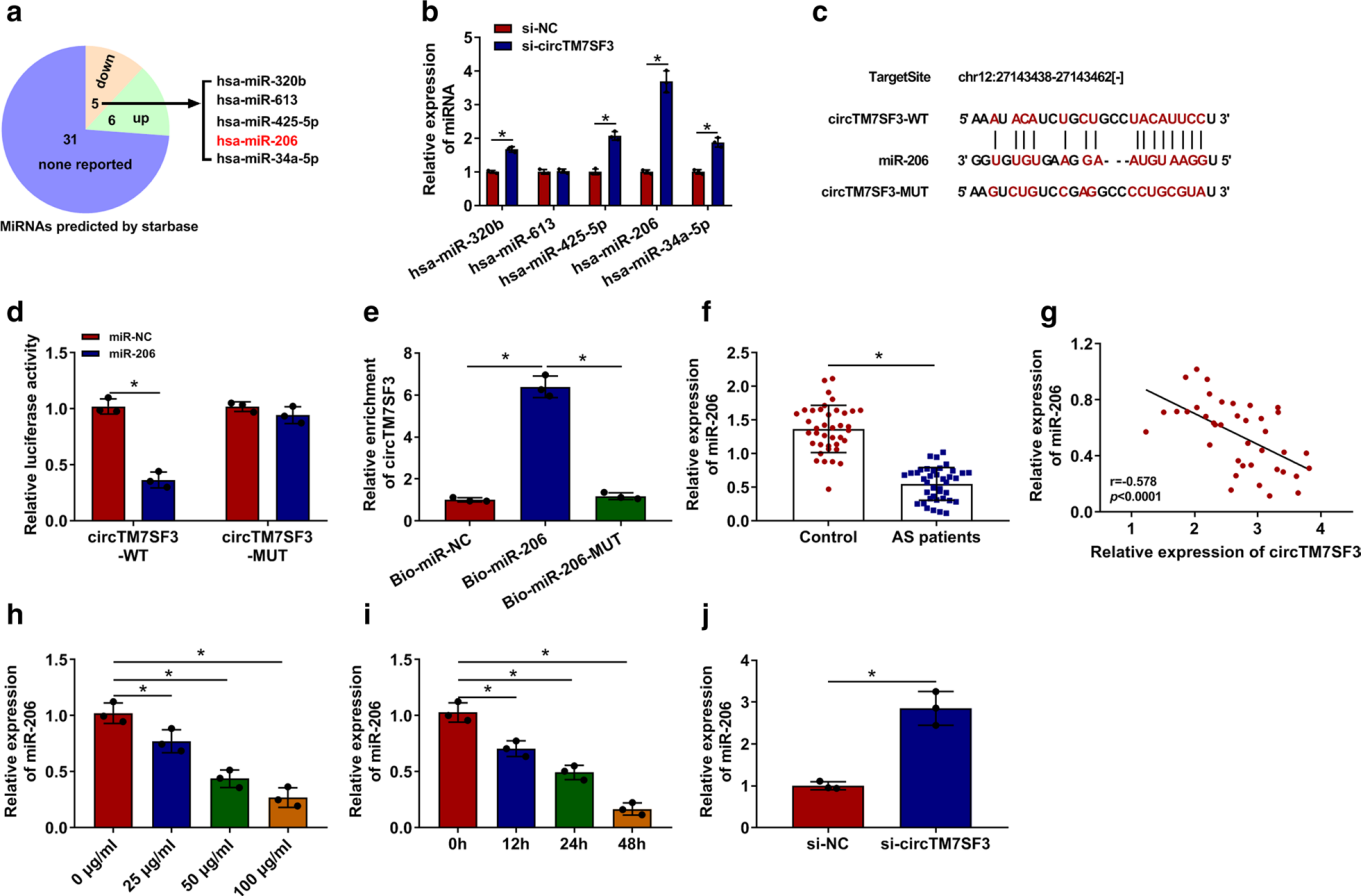
CircTM7SF3 directly interacts with miR-206 in THP-1-derived macrophages**. a** The miRNA targets of circTM7SF3 were predicted by starbase software. Among these putative miRNA targets, five miRNAs (miR-320b, miR-613, miR-425-5p, miR-206 and miR-34a-5p) have been reported to be down-regulated in AS. **b** The expression of miR-320b, miR-613, miR-425-5p, miR-206 and miR-34a-5p in differentiated THP-1 cells transfected with si-NC or si-circTM7SF3 was measured by qRT-PCR (*n* = 3). The results were analyzed using Student’s *t*-test. **c** The target sequence of miR-206 in circTM7SF3 that predicted by starbase software was shown. Also, the mutant binding sites with miR-206 in circTM7SF3 were also shown. **d** Dual-luciferase reporter assay was performed to verify the interaction between miR-206 and circTM7SF3 in THP-1 cells. THP-1 cells (*n* = 3) were co-transfected with miR-NC or miR-206 and circTM7SF3 wild-type reporter plasmid (circTM7SF3-WT) or circTM7SF3 mutant reporter plasmid (circTM7SF3-MUT). The results were analyzed using Student’s *t*-test. **e** RNA-pull down assay was carried out to test the target interaction between miR-206 and circTM7SF3 in THP-1 cells (*n* = 3). The results were assessed by ANOVA followed by Tukey’s test. **f** MiR-206 level in the serum of Control group (*n* = 39) and AS patients (*n* = 39) was analyzed by qRT-PCR. The results were analyzed using Student’s *t*-test. **g** The linear correlation between the expression of circTM7SF3 and miR-206 in the serum of AS patients (*n* = 39) was analyzed using Spearman’s correlation coefficient. **h** and **i** MiR-206 level in THP-1 cells (*n* = 3) treated with different doses or times of ox-LDL was analyzed by qRT-PCR. The results were assessed by ANOVA followed by Tukey’s test. **j** MiR-206 level was examined in THP-1 cells (*n* = 3) transfected with si-NC or si-circTM7SF3 by qRT-PCR. The results were analyzed using Student’s *t*-test. *: *P* < 0.05

Starbase software was used to seek the possible miRNA targets of circTM7SF3 (Fig. 3a). Among these candidate miRNA targets, five miRNAs, including miR-320b, miR-613, miR-425-5p, miR-206 and miR-34a-5p, have been reported to be down-regulated in AS, which exhibited to an opposite tendency to circTM7SF3, thus we focused on these five miRNAs. THP-1-derived macrophages were transfected with si-NC or si-circTM7SF3 for 24 h, and we found that the expression of miR-320b, miR-425-5p, miR-206 and miR-34a-5p was up-regulated with the interference of circTM7SF3, especially the miR-206 (Fig. 3b), thus we concentrated on the potential interaction between miR-206 and circTM7SF3. The putative binding sites between miR-206 and circTM7SF3 were shown in Fig. 3c. To test whether miR-206 could bind to circTM7SF3 in THP-1-derived macrophages, we conducted dual-luciferase reporter assay and RNA-pull down assay. THP-1-derived macrophages were co-transfected with miR-NC or miR-206 and circTM7SF3-WT or circTM7SF3-MUT. Compared with miR-NC and circTM7SF3-WT co-transfected group, luciferase activity was markedly reduced in circTM7SF3-WT group when miR-206 was overexpressed (Fig. 3d). Furthermore, the luciferase activity was un-affected in circTM7SF3-MUT group when co-transfected with miR-NC or miR-206 (Fig. 3d), suggesting that miR-206 bound to circTM7SF3 via the predicted sites. MiR-206 was biotinylated to pull down its associated molecules. As shown in Fig. 3e, circTM7SF3 was enriched in Bio-miR-206 precipitation complex, which further verified the interaction between miR-206 and circTM7SF3. The expression of miR-206 in AS patients and healthy volunteers was explored. There was a significant reduction in miR-206 expression in the serum samples of AS patients (*n* = 39) compared with healthy volunteers (*n* = 39) (Fig. 3f). Given the opposite expression tendency between miR-206 and circTM7SF3, we analyzed the linear correlation between the two molecules. CircTM7SF3 expression was negatively correlated with the level of miR-206 (Fig. 3g; *P*<0.0001). MiR-206 level was decreased with ox-LDL treatment in THP-1-derived macrophages in a dose- or time-dependently manner (Fig. 3h and i). MiR-206 level was notably increased with the interference of circTM7SF3 in THP-1-derived macrophages (Fig. 3j), suggesting the negative regulatory relationship between miR-206 and circTM7SF3.

### Si-circTM7SF3-mediated effects in ox-LDL-induced THP-1-derived macrophages are partly counteracted by the addition of anti-miR-206

**Fig. 4:**
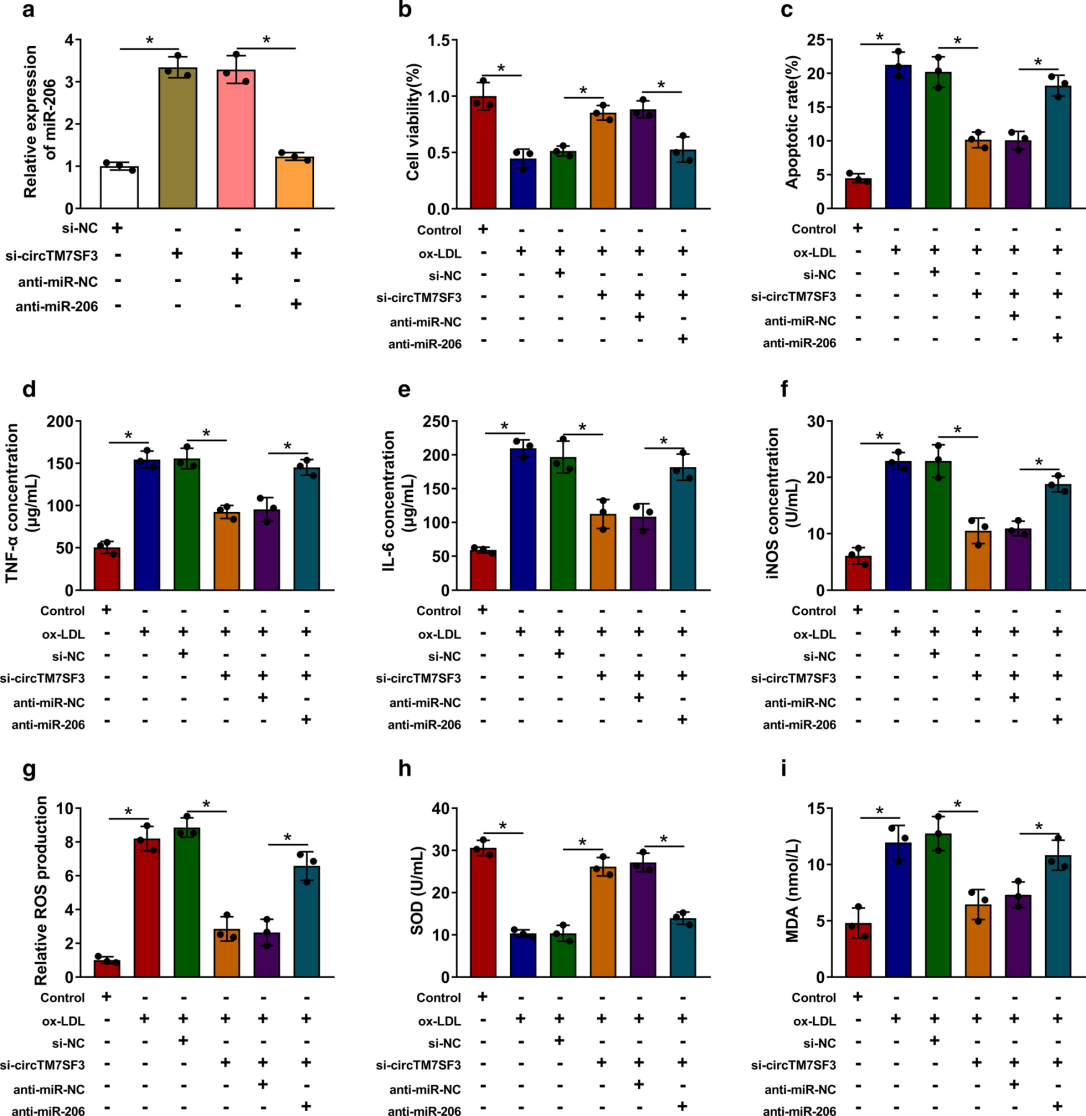
Si-circTM7SF3-mediated effects in ox-LDL-induced THP-1-derived macrophages are partly counteracted by the addition of anti-miR-206. **a** THP-1 cells (*n* = 3) were transfected with si-circTM7SF3 alone or co-transfected with si-circTM7SF3 and anti-miR-206. MiR-206 expression was detected in THP-1 cells by qRT-PCR. The results were assessed by ANOVA followed by Tukey’s test. **b**-**i** THP-1 cells were divided into six groups: Control, ox-LDL, ox-LDL + si-NC, ox-LDL + si-circTM7SF3, ox-LDL + si-circTM7SF3 + anti-miR-NC or ox-LDL + si-circTM7SF3 + anti-miR-206. The results in B-I were assessed by ANOVA followed by Tukey’s test. **b** Cell viability was analyzed by MTT assay (*n* = 3). **c** The apoptotic rate was measured by flow cytometry (*n* = 3). **d** and **e** The levels of inflammation-related cytokines were detected by ELISA kits (*n* = 3). (F-I) The levels of iNOS, ROS, SOD and MDA in THP-1 cells were measured by their corresponding kits (*n* = 3). *: *P* < 0.05

MiR-206 level was elevated in circTM7SF3 silencing group, and the co-transfection with anti-miR-206 decreased the expression of miR-206 in THP-1-derived macrophages (Fig. 4a). We conducted rescue experiments using similar treatments in ox-LDL-induced THP-1-derived macrophages to test if circTM7SF3 functioned partly through targeting miR-206. The protective effect of si-circTM7SF3 in the viability of ox-LDL-induced THP-1-derived macrophages was ameliorated by the introduction of anti-miR-206 (Fig. 4b). Also, the apoptosis was inhibited in ox-LDL and si-circTM7SF3 group, and miR-206 silencing promoted the apoptosis of THP-1-derived macrophages again (Fig. 4c). The release of pro-inflammatory cytokines was suppressed in circTM7SF3 silencing group in ox-LDL-induced THP-1-derived macrophages, and the addition of anti-miR-206 further enhanced the concentrations of TNF-α and IL-6 (Fig. 4d and e). The levels of iNOS, ROS and MDA were reduced in si-circTM7SF3 and ox-LDL group, while the expression of these molecules was partly up-regulated with the co-transfection of anti-miR-206 in THP-1-derived macrophages (Fig. 4f, g and i). The level of SOD exhibited a reverse phenomenon to iNOS, ROS or MDA (Fig. 4h), demonstrating that circTM7SF3 interference suppressed the oxidative stress of ox-LDL-induced THP-1-derived macrophages partly through elevating miR-206 level.

### ASPH is a direct target of miR-206 in THP-1-derived macrophages

**Fig. 5:**
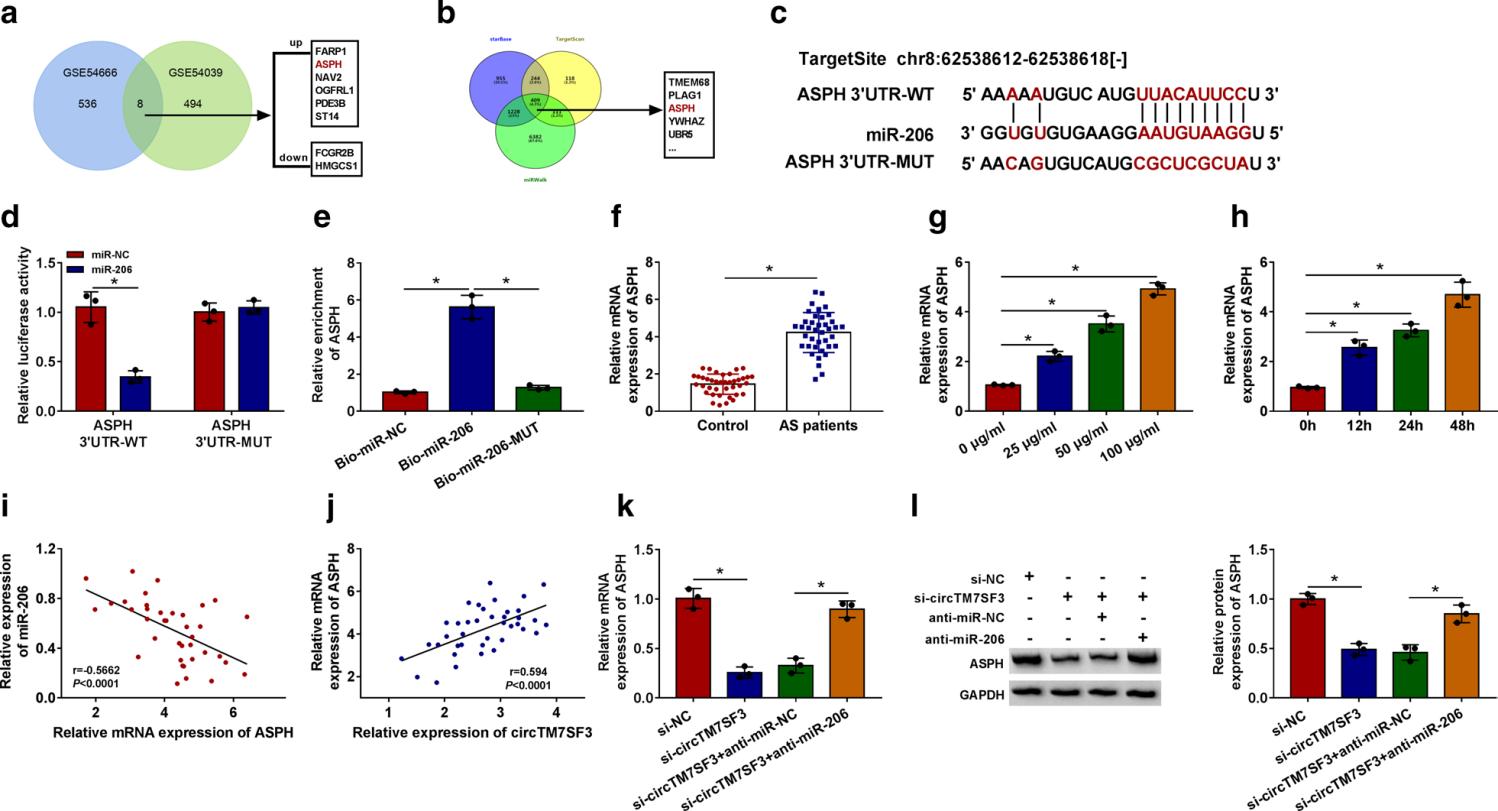
ASPH is a direct target of miR-206 in THP-1-derived macrophages. **a** The overlapped eight genes in GSE54666 and GSE54039 in ox-LDL-induced THP-1 cells were shown. Among these eight genes, six genes (including ASPH) were up-regulated, while two genes were down-regulated. **b** The targets of miR-206 were analyzed using starbase, miRWalk and TargetScan softwares. There were 409 genes (including ASPH) that simultaneously predicted to be targets of miR-206 by these three bioinformatic softwares. **c** The putative binding sites between miR-206 and ASPH predicted by starbase software were shown. **d** and **e** The target relationship between miR-206 and ASPH was evaluated by dual-luciferase reporter assay and RNA-pull down assay (*n* = 3). The results in D were evaluated by Student’s *t*-test, while the results in E were assessed by ANOVA followed by Tukey’s test. **f** ASPH mRNA level in the serum samples of control healthy volunteers (*n* = 39) and AS patients (*n* = 39) was detected by qRT-PCR. The results were analyzed using Student’s *t*-test. **g** and **h** After exposing to ox-LDL for different doses or treatment times, ASPH mRNA level was measured by qRT-PCR assay (*n* = 3). The results were assessed by ANOVA followed by Tukey’s test. (I and J) The linear relation between the levels of ASPH mRNA and miR-206 or circTM7SF3 was analyzed by Spearman’s correlation coefficient. **k** and **l** THP-1 cells were treated with si-NC, si-circTM7SF3, si-circTM7SF3 + anti-miR-NC or si-circTM7SF3 + anti-miR-206. The mRNA and protein levels of ASPH in THP-1 cells (*n* = 3) were detected by qRT-PCR and Western blot assay. The protein bands shown in Fig. 5L have been cropped. The original Western blot data in Fig. 5L could be find in Additional file 2. The results were assessed by ANOVA followed by Tukey’s test. *: *P* < 0.05

We analyzed the aberrantly expressed molecules in GSE54666 and GSE54039 datasets, and eight genes were consistently reported to be up-regulated or down-regulated in ox-LDL-induced THP-1-derived macrophages in two datasets (Fig. 5a). ASPH was found to be up-regulated after ox-LDL treatment in both datasets (Fig. 5a). Meanwhile, we predicted the downstream genes of miR-206 through using three bioinformatic softwares, including starbase, miRWalk and TargetScan softwares. A total of 409 genes were predicted to be putative targets of miR-206 by three softwares (Fig. 5b). In view of the expression pattern and the possible interaction with miR-206, we aimed to test the interaction between miR-206 and ASPH using dual-luciferase reporter assay and RNA-pull down assay. The complementary sites between miR-206 and ASPH predicted by starbase software were shown in Fig. 5c. Luciferase activity was dramatically reduced in miR-206 and ASPH 3’UTR-WT group compared with miR-NC and ASPH 3’UTR-WT group (Fig. 5d), proving the direct interaction between these two genes in THP-1-derived macrophages. The results of RNA-pull down assay suggested that ASPH mRNA was most enriched in Bio-miR-206 group than that in Bio-miR-NC or Bio-miR-206-MUT group (Fig. 5e), which further confirmed the interaction between miR-206 and ASPH. The expression of ASPH mRNA was significantly enhanced in the serum samples of AS patients (*n* = 39) in comparison with that in healthy volunteers (*n* = 39) (Fig. 5f). The mRNA level of ASPH was elevated in THP-1-derived macrophages with ox-LDL treatment in a dose- or time-dependent manner (Fig. 5g and h). ASPH expression was negatively correlated with miR-206 level (*P*<0.0001), while there was a positive correlation between the expression of ASPH and circTM7SF3 (*P*<0.0001) (Fig. 5i and j). After verifying the interaction between miR-206 and circTM7SF3 or ASPH, we aimed to test the regulatory relationship among these three genes in THP-1-derived macrophages. As shown in Fig. 5k and l, circTM7SF3 silencing down-regulated the mRNA and protein levels of ASPH, and its mRNA and protein levels were up-regulated in si-circTM7SF3 and anti-miR-206 co-transfected group, suggesting that circTM7SF3 silencing reduced ASPH expression partly via up-regulating miR-206 in THP-1-derived macrophages.

### MiR-206 overexpression-mediated influences in ox-LDL-induced THP-1-derived macrophages are partly overturned by ASPH accumulation

**Fig. 6:**
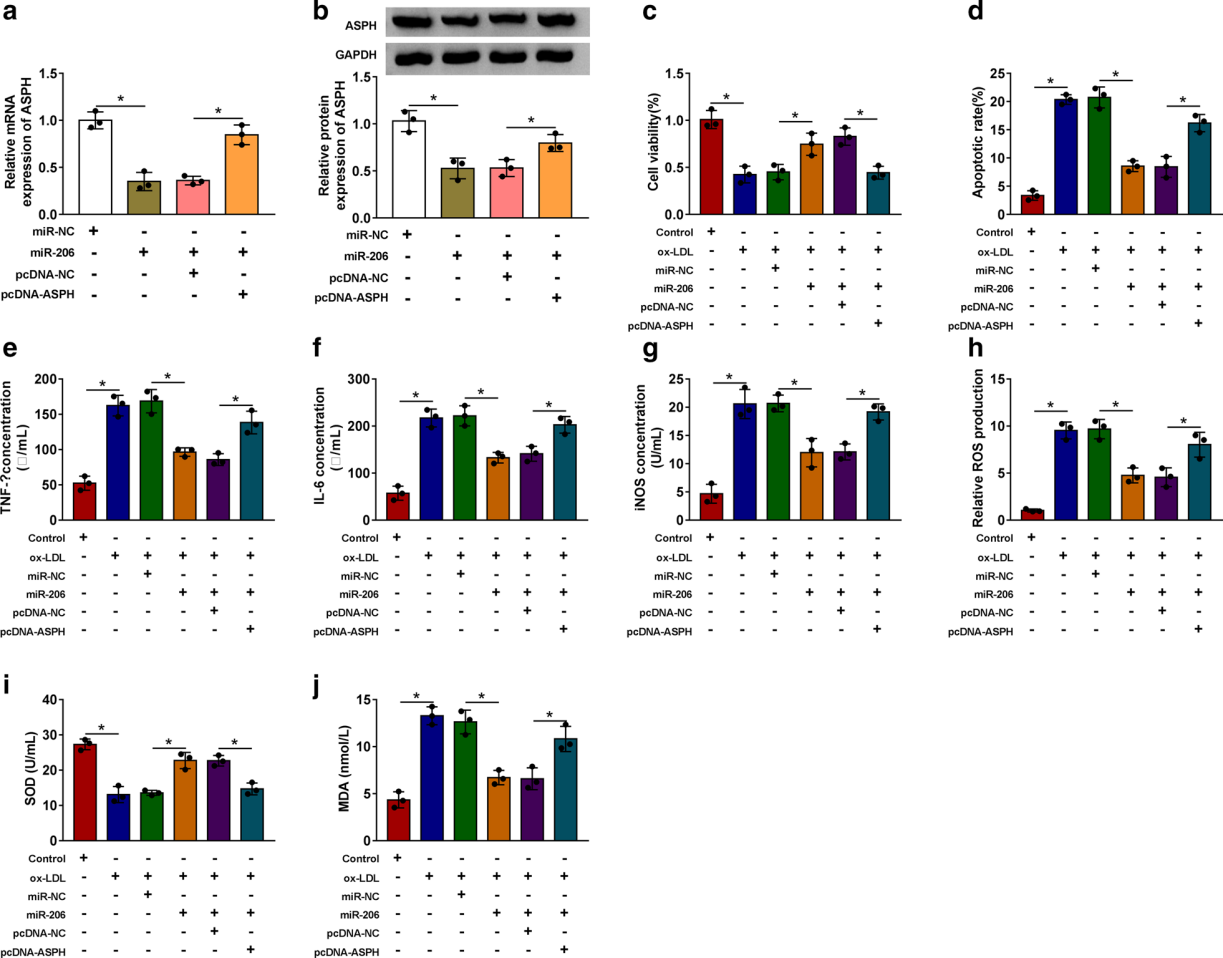
MiR-206 overexpression-mediated influences in ox-LDL-induced THP-1-derived macrophages are partly overturned by ASPH accumulation. **a** and **b** THP-1 cells (*n* = 3) were transfected with miR-NC, miR-206, miR-206 + pcDNA-NC or miR-206 + pcDNA-ASPH. qRT-PCR and Western blot assay were used to detect the mRNA and protein expression of ASPH. The protein bands shown in Fig. 6B have been cropped. The original Western blot data in Fig. 6B could be find in Additional file 2. The results were assessed by ANOVA followed by Tukey’s test. **c**-**j** THP-1 cells were treated with ox-LDL, ox-LDL + miR-NC, ox-LDL + miR-206, ox-LDL + miR-206 + pcDNA-NC or ox-LDL + miR-206 + pcDNA-ASPH. The results in **c**-**j** were assessed by ANOVA followed by Tukey’s test. **c** MTT assay was conducted to assess the cell viability of transfected THP-1 cells (*n* = 3). **d** The apoptosis of THP-1 cells (*n* = 3) was measured by flow cytometry. (E and F) ELISA kits were used to detect the concentrations of TNF-α and IL-6 (*n* = 3). **g**-**j** The levels of iNOS, ROS, SOD and MDA were detected by their matching kits (*n* = 3). *: *P* < 0.05

We conducted rescue experiments through transfecting miR-206 alone or together with pcDNA-ASPH into THP-1-derived macrophages to test if miR-206 exerted its functions partly by down-regulating ASPH. MiR-206 accumulation reduced the mRNA and protein enrichment of ASPH, and its mRNA and protein levels were largely rescued by the addition of ASPH overexpression plasmid (Fig. 6a and b). ox-LDL-induced reduction of cell viability and promotion of apoptosis were largely attenuated by the overexpression of miR-206 (Fig. 6c and d), and these findings further confirmed that miR-206 protected THP-1-derived macrophages against ox-LDL-induced injury. Furthermore, ASPH overexpression ameliorated the protective role of miR-206 in ox-LDL-induced THP-1-derived macrophages again (Fig. 6c and d). MiR-206 overexpression diminished the inflammatory response and oxidative stress of THP-1-derived macrophages (Fig. 6e-j). The addition of ASPH overexpression plasmid promoted the release of TNF-α and IL-6 (Fig. 6e, f). Through analyzing the levels of iNOS, ROS and MDA and SOD in six groups, we found that miR-206-mediated suppression in the oxidative stress of ox-LDL-induced THP-1-derived macrophages was overturned by the addition of pcDNA-ASPH (Fig. 6g-j).

## Discussion

We found that circTM7SF3 level was elevated with ox-LDL treatment in THP-1-derived macrophages, and it was also up-regulated in the serum of AS patients compared with healthy controls. CircTM7SF3/miR-206/ASPH signal axis was identified in this study for the first time. CircTM7SF3 promoted ox-LDL-induced cell injury in AS cell model through increasing ASPH expression via sponging miR-206.

AS is a polygenic and multifactorial inflammatory disorder [26]. Macrophages are identified as major contributors for the initiation and progression of AS [27]. In the current study, we established AS cell model through exposing THP-1-derived macrophages to ox-LDL in vitro. CircTM7SF3 level was found to be notably increased in AS patients in contrast to that in healthy volunteers. Besides, ox-LDL markedly enhanced circTM7SF3 level in a dose- and time-dependent manner in THP-1-derived macrophages. Through silencing circTM7SF3 in ox-LDL-treated AS cell model, we found that ox-LDL-mediated injury in THP-1-derived macrophages was largely ameliorated, suggesting that ox-LDL promoted the apoptosis, inflammation and oxidative stress and inhibited the viability of THP-1-derived macrophages partly through up-regulating circTM7SF3 level. The results of this study on THP-1-derived macrophages were further confirmed on a hMDMs AS cell model. We observed that ox-LDL also accelerated the inflammatory response of differentiated hMDMs via up-regulating circTM7SF3.

MiR-206 was identified as a tumor suppressor in many types of cancers. For instance, miR-206 hampered the proliferation and invasion of thyroid cancer cells through targeting RAP1B [14]. MiR-206 restrained the proliferation and motility of cervical cancer cells through targeting BAG3 [28]. MiR-206 has also been implicated in the regulatory mechanism of cardiovascular injury. Hu et al. claimed that lncRNA UCA1 promoted ox-LDL-induced injury in THP-1 cells through sponging miR-206 [19]. Accumulating studies have reported that circRNAs functioned through targeting miRNAs [29–31]. For instance, circITGA7 hampered the progression of colorectal cancer through sponging miR-3187-3p, thus elevating ASXL1 level [32]. Circ_0003204 suppressed the proliferation, migration and tube formation of endothelial cells in AS through regulating miR-370-3p [33]. Here, the direct interaction between circTM7SF3 and miR-206 was confirmed. MiR-206 level was negatively regulated by ox-LDL or circTM7SF3 in THP-1-derived macrophages. Further studies suggested that ox-LDL induced the injury of THP-1-derived macrophages through targeting circTM7SF3/miR-206 signal axis.

ASPH is a member of α-ketoglutarate-dependent dioxygenase family [34]. ASPH is highly expressed in embryogenesis, and it accelerates cellular migration in organ development [35, 36]. ASPH was involved in the regulation of a few cancers. Zou et al. found that the hydroxylase activity of ASPH accelerated the epithelial-to-mesenchymal transition of hepatocellular carcinoma cells [20]. Chen et al. demonstrated that miR-135a exerted its anti-tumor role in endometrial cancer through targeting ASPH [21]. Here, ASPH was found as a direct target of miR-206 in THP-1-derived macrophages. CircTM7SF3 elevated the expression of ASPH through sponging miR-206 in THP-1-derived macrophages. Furthermore, rescue experiments revealed that miR-206 exhibited a protective role in ox-LDL-induced THP-1-derived macrophages through down-regulating ASPH level.

## Limitations

Human acute monocytic leukemia cell line THP-1 was used for most of the experiments. Because THP-1 was a tumorigenic cell line, the results of experiments from AS cell model using ox-LDL-induced THP-1-derived macrophages might be different from AS cell model using ox-LDL-induced primary macrophages.

## Conclusion

**Fig. 7:**
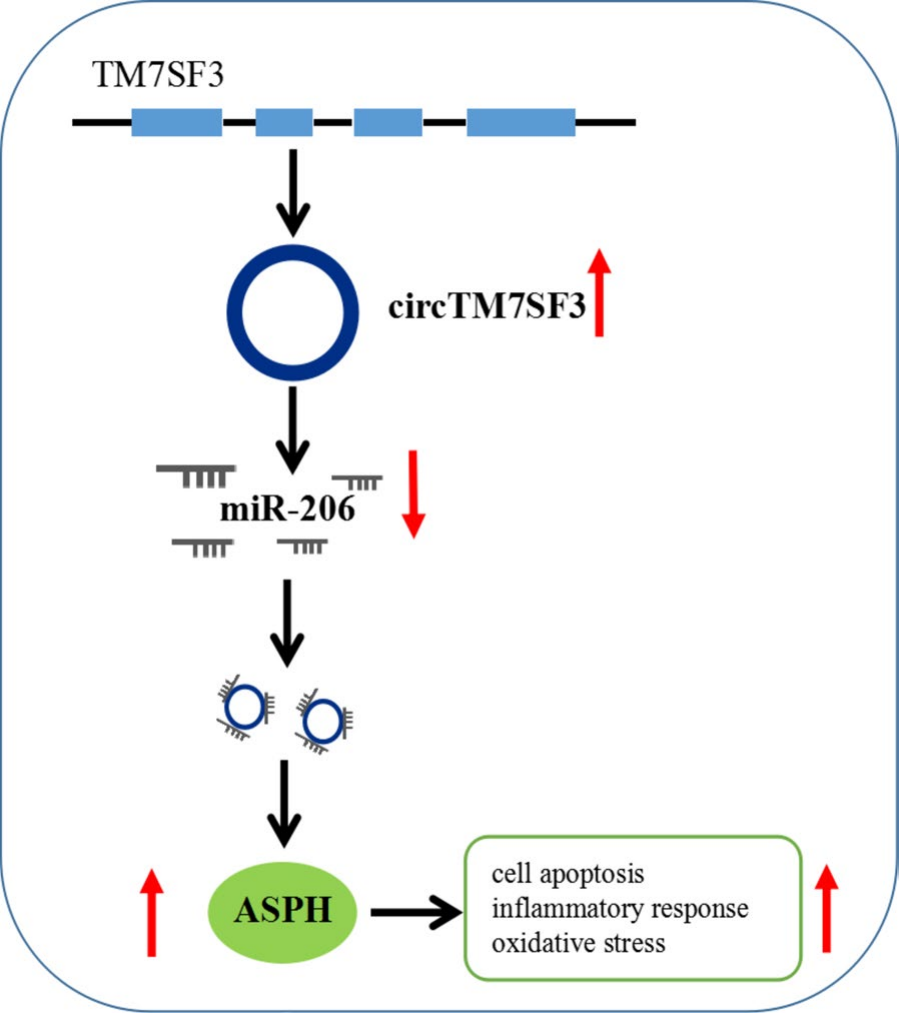
A schematic of the working mechanism of circTM7SF3 in ox-LDL-induced AS cell model

Taken together, circTM7SF3 was a circular RNA generated from TM7SF3 gene that stably existed in THP-1-derived macrophages. CircTM7SF3 contributed to ox-LDL-induced apoptosis, inflammatory response and oxidative stress through targeting miR-206, thus causing the increase of ASPH expression (Fig. 7). The identification of circTM7SF3/miR-206/ASPH signal axis disclosed a novel insight into the role of circTM7SF3 in AS progression, providing promising therapeutic target for AS.

## Supplementary Information

**Additional file 1**.** Figure 1**: ox-LDL induces the inflammatory response of differentiated human monocyte-derived macrophages (hMDMs) partly via up-regulating circTM7SF3. (A-C) Differentiated hMDMs were divided into four groups: Control, ox-LDL, ox-LDL + si-NC and ox-LDL + si-circTM7SF3. The results in A-C were assessed by ANOVA followed by Tukey’s test. (A) The relative level of circTM7SF3 in THP-1 cells (*n* = 3) was measured by qRT-PCR. (B and C) The production of TNF-α and IL-6 was analyzed using ELISA kits (*n* = 3). *: *P* < 0.05.

**Additional file 2.** Original Western blot data.

## Data Availability

The datasets generated during the current study are not publicly available yet, due to privacy concerns and ongoing additional research. Data can be made available for peer review on reasonable request through contacting the corresponding author.

## Abbreviations

AS: Atherosclerosis
circTM7SF3: Circular RNA transmembrane 7 superfamily member 3
qRT-PCR: Quantitative real-time polymerase chain reaction
miR-206: microRNA-206
ELISA: Enzyme-linked immunosorbent assay
ox-LDL: Oxidized low-density lipoprotein
circRNAs: Circular RNAs
VSMCs: Vascular smooth muscle cells
lncRNA: Long non-coding RNA
ASPH: Aspartyl (asparaginyl) β-hydroxylase
GAPDH: Glyceraldehyde-3-phosphate dehydrogenase
si-NC: siRNA negative control
