# Supplementary figures and images for: CircTM7SF3 contributes to oxidized low-density lipoprotein-induced apoptosis, inflammation and oxidative stress through targeting miR-206/ASPH axis in atherosclerosis cell model in vitro
Source: BMC Cardiovasc Disord. 2021 Feb 2;21:51. doi: 10.1186/s12872-020-01800-x (PMC7852086; doi:10.1186/s12872-020-01800-x)

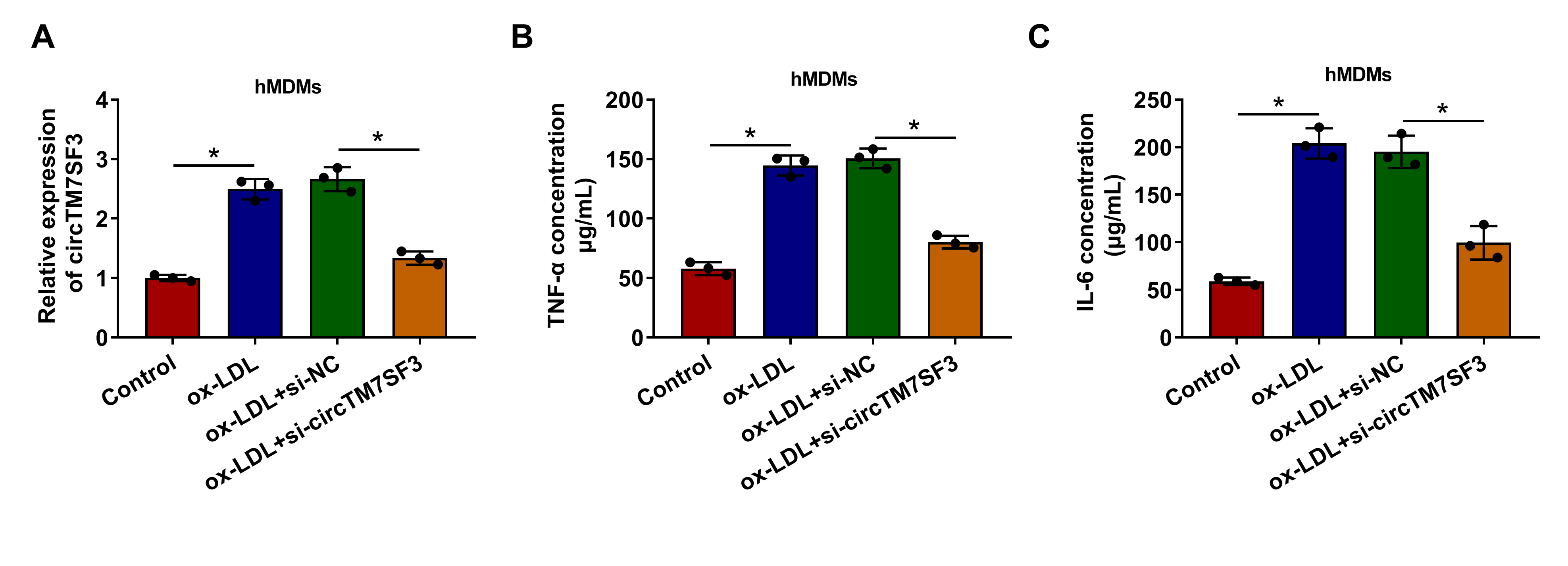

Supplement: Supplementary file 1 — Additional file 1. Figure 1: ox-LDL induces the inflammatory response of differentiated human monocyte-derived macrophages (hMDMs) partly via up-regulating circTM7SF3. (A-C) Differentiated hMDMs were divided into four groups: Control, ox-LDL, ox-LDL + si-NC and ox-LDL + si-circTM7SF3. The results in A-C were assessed by ANOVA followed by Tukey’s test. (A) The relative level of circTM7SF3 in THP-1 cells (n = 3) was measured by qRT-PCR. (B and C) The production of TNF-α and IL-6 was analyzed using ELISA kits (n = 3). *: P < 0.05. [file 12872_2020_1800_MOESM1_ESM.tif]
