# Supplementary material for: CircTM7SF3 contributes to oxidized low-density lipoprotein-induced apoptosis, inflammation and oxidative stress through targeting miR-206/ASPH axis in atherosclerosis cell model in vitro
Source: BMC Cardiovasc Disord. 2021 Feb 2;21:51. doi: 10.1186/s12872-020-01800-x (PMC7852086; doi:10.1186/s12872-020-01800-x)
Supplement: Supplementary file 2 — Additional file 2. Original Western blot data. [file 12872_2020_1800_MOESM2_ESM.pdf]

Figure 5. ASPH is a direct target of miR-206 in THP-1 cells

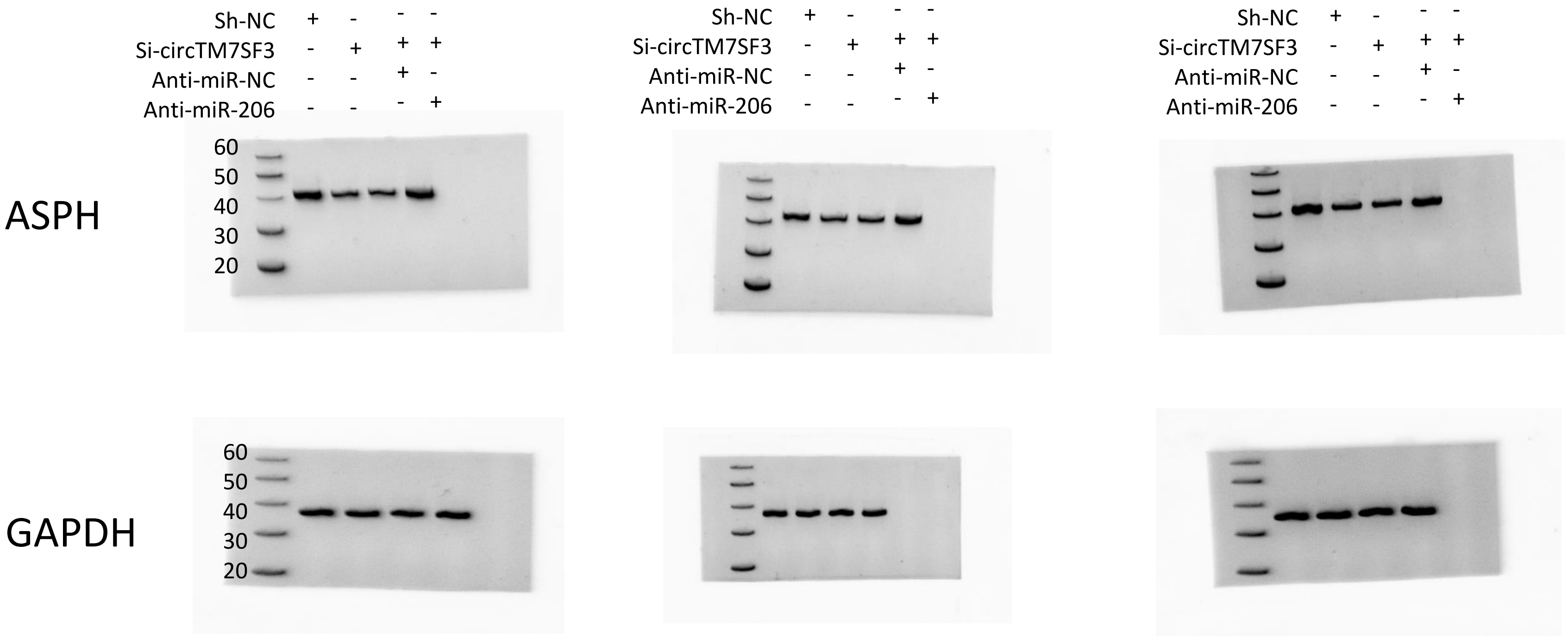

(L) THP-1 cells were treated with si-NC, si-circTM7SF3, si-circTM7SF3 + anti-miR-NC or si-circTM7SF3 + anti-miR-206. The mRNA and protein abundance of ASPH in THP-1 cells was detected by Western blot assay.

Figure 6. MiR-206 overexpression-mediated influences in ox-LDL-induced THP-1 cells are partly overturned by ASPH accum ulation .

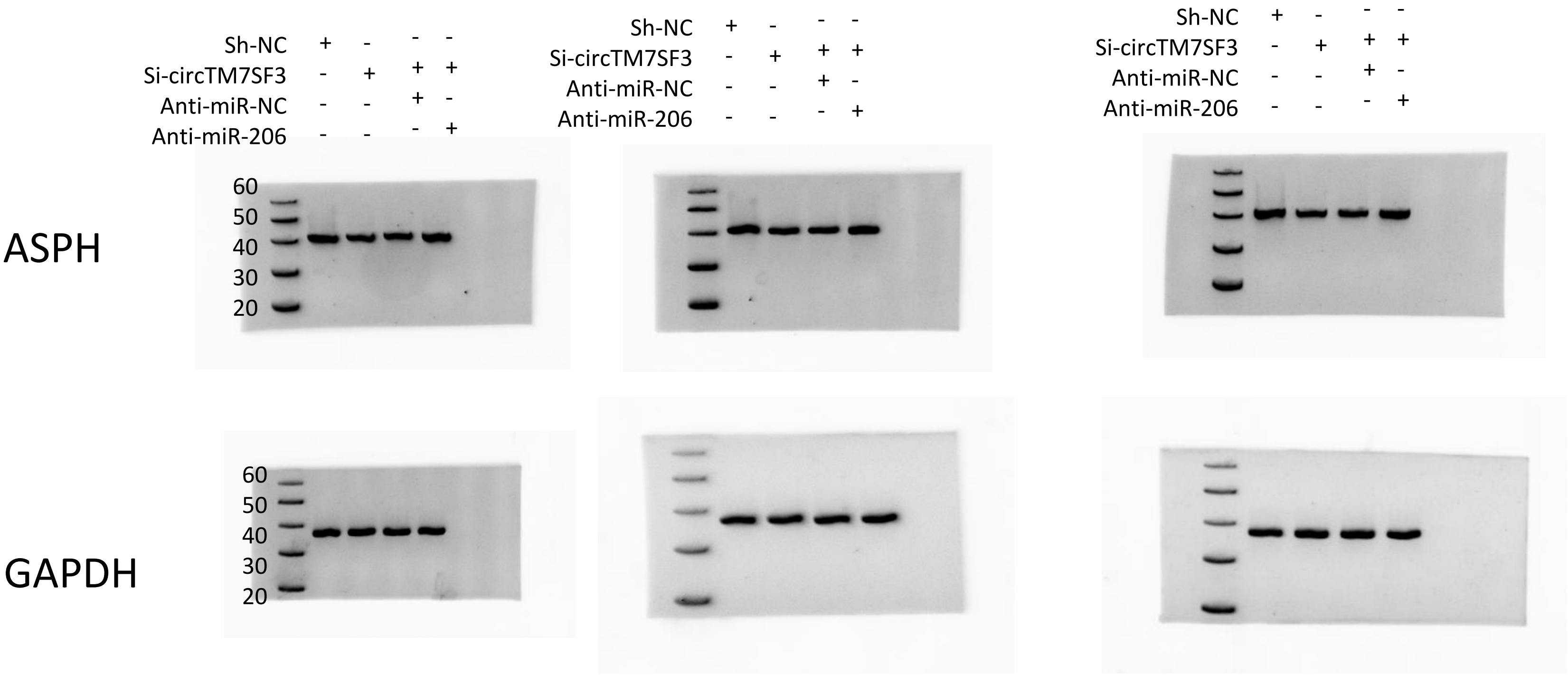

(B) THP-1 cells were transfected with miR-NC, miR-206, miR-206 + pcDNA-NC or miR-206 + pcDNA-ASPH. Western blot assay were used to detect the mRNA and protein expression of ASPH.
